# Activity-dependent disruption of intersublaminar spaces and ABAKAN expression does not impact functional on and off organization in the ferret retinogeniculate system

**DOI:** 10.1186/1749-8104-6-7

**Published:** 2011-03-14

**Authors:** Colenso M Speer, Chao Sun, Barbara Chapman

**Affiliations:** 1 Center for Neuroscience, University of California, Davis, Davis, California, 95618 USA; 2 Department of Chemistry and Chemical Biology, Harvard University, 12 Oxford Street, Cambridge, MA 02138, USA; 3 Department of Neurobiology, Physiology, and Behavior, University of California, Davis, Davis, California, 95616 USA

## Abstract

In the adult visual system, functionally distinct retinal ganglion cells (RGCs) within each eye project to discrete targets in the brain. In the ferret, RGCs encoding light increments or decrements project to independent On and Off sublaminae within each eye-specific layer of the dorsal lateral geniculate nucleus (dLGN). Here we report a manipulation of retinal circuitry that alters RGC action potential firing patterns during development and eliminates the anatomical markers of segregated On and Off sublaminae in the LGN, including the intersublaminar spaces and the expression of a glial-associated inhibitory molecule, ABAKAN, normally separating On and Off leaflets. Despite the absence of anatomically defined On and Off sublaminae, electrophysiological recordings in the dLGN reveal that On and Off dLGN cells are segregated normally. These data demonstrate a dissociation between normal anatomical sublamination and segregation of function in the dLGN and call into question a purported role for ABAKAN boundaries in the developing visual system.

## Background

During visual system development, retinal ganglion cells (RGCs) within each eye form precise parallel pathways to central visual targets. In the ferret, two broad classes of RGCs responsive to increments and decrements of light in their receptive field centers (On and Off RGCs) form discrete sublaminae within each eye-specific layer of the retinogeniculate pathway [1-3].

Between postnatal day 10 (P10) and P25, On and Off retinogeniculate afferents segregate anatomically [2,3] and are separated by an emerging margin of glial cells and extracellular matrix [4-7]. The glial territories separating eye-specific and On and Off layers in the dorsal lateral geniculate nucleus (dLGN) contain a keratin sulfate proteoglycan, ABAKAN, which is expressed in a developmentally regulated pattern coinciding with On and Off sublaminar development [4]. Based on expression pattern, developmental timing of expression, and action as a repulsive signaling molecule [4,8,9], ABAKAN has been hypothesized to act as a restrictive boundary signal that contributes to the establishment and/or maintenance of RGC targeting to On and Off layers of the dLGN [4]. However, a direct role for this molecule in the development of segregated On and Off sublaminae has not been demonstrated.

The refinement of normal On and Off sublaminae in the ferret dLGN is known to depend on spontaneous activity occurring in the developing retinogeniculate pathway. Blockade of spontaneous retinal activity [10] or postsynaptic readout of spontaneous retinal input [11,12] each leads to a disruption of On and Off retinogeniculate projections. These findings implicate spontaneous activity in the development of anatomically segregated On and Off sublaminae, but leave open the issue of whether there is a functional consequence of abolishing the intersublaminar space and disrupting the anatomical specificity of On and Off RGC targeting.

In order to examine the contributions of spontaneous retinal activity to On and Off sublaminar refinement, we developed a novel immunotoxin (Ferret VAChT-SAP - the vesicular acetylcholine transporter (VAChT) protein conjugated to Saporin toxin) targeting cholinergic starburst amacrine cells (SACs) of the ferret retina in order to ablate this population and disrupt spontaneous retinal activity during sublaminar segregation (see Additional file 1 for a timeline of the experimental manipulation). Previous work in our lab used a similar approach to evaluate contributions of spontaneous retinal activity to eye-specific retinogeniculate segregation [13]. In that study, a different immunotoxin based on a mouse *VAChT *gene sequence was used. In contrast, here we employ a ferret-specific VAChT-SAP immunotoxin based on a cloned ferret *VAChT *gene sequence (see Materials and methods). Immunotoxin treatment disrupts spontaneous retinal activity and prevents formation of the intersublaminar space between On and Off retinogeniculate afferents, including expression of ABAKAN between On and Off leaflets in the dLGN. Despite these anatomical defects, On and Off responses recorded in the dLGNs of adult ferrets previously treated with Ferret VAChT-SAP exhibit center-surround receptive fields of a single center sign and are distributed normally relative to controls. These data demonstrate that functional On and Off channels develop normally in the absence of clear anatomical hallmarks of On and Off segregation and cast doubt on a role for ABAKAN in the establishment of functional On and Off sublaminae in the ferret dLGN.

## Results

### Ablation of starburst amacrine cells by Ferret VAChT-SAP

**Figure 1:**
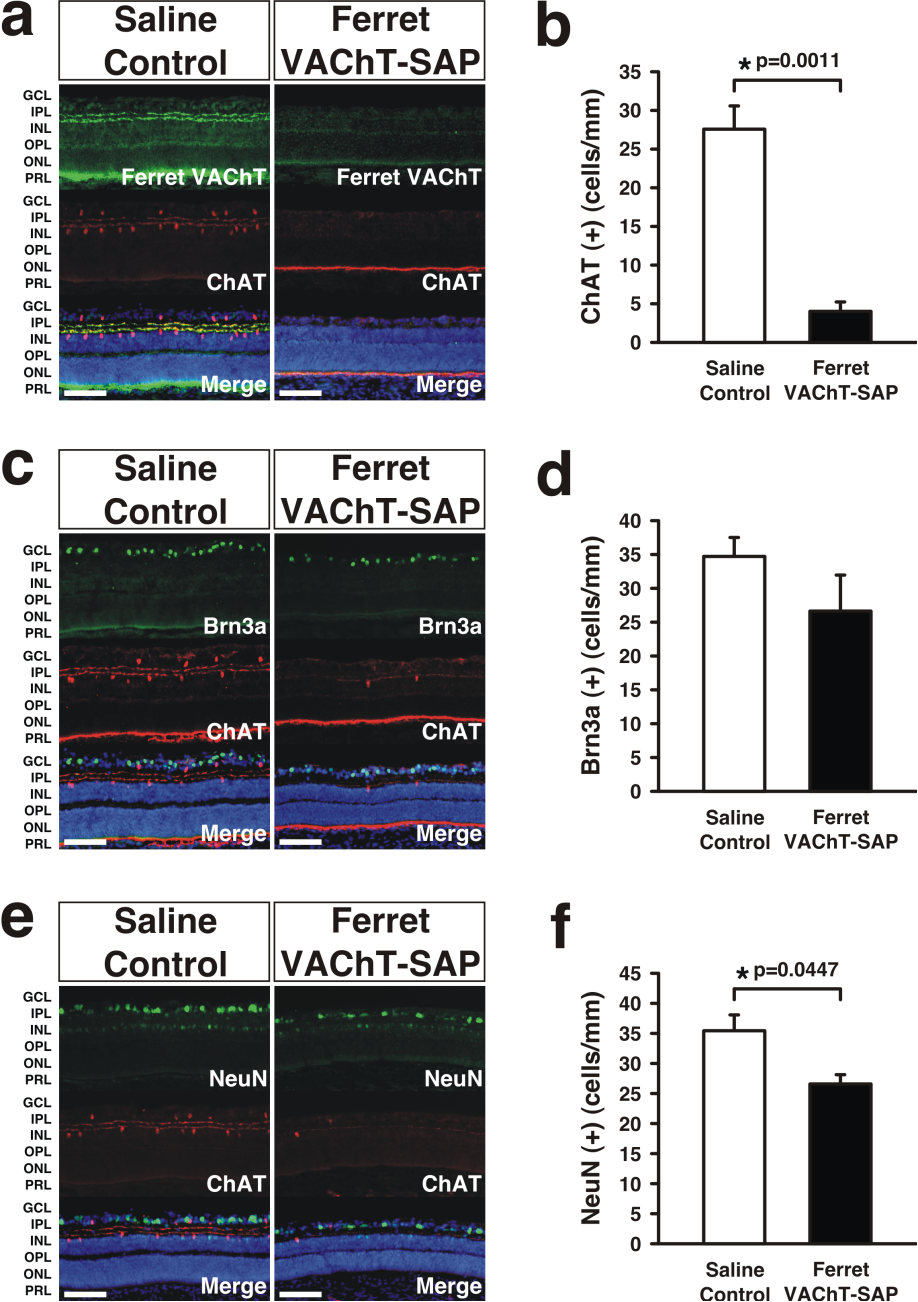
**Effects of Ferret VAChT-SAP treatment on starburst amacrine and retinal ganglion cell ablation**. **(A,B) **Treatment with Ferret VAChT-SAP leads to ablation of ChAT(+) starburst amacrine cells (SACs) and loss of vesicular acetylcholine transporter (VAChT) expression. **(C-F) **Brn3a(+) and NeuN(+) retinal ganglion cells (RGCs) are also ablated, although to a lesser degree than SACs, by Ferret VAChT-SAP treatment ((C,E) photomicrographs; (D,F) quantification). Data reflect means ± standard errors of the mean. Statistics reflect two-tailed *P*-values calculated from independent two sample *t*-tests. 'Merge' panels are counterstained with DAPI to reveal cytoarchitecture. GCL, ganglion cell layer; IPL, inner plexiform layer, INL, inner nuclear layer; OPL, outer plexiform layer; ONL, outer nuclear layer; PRL, photoreceptor layer. Scale bars: 100 μm.

In order to investigate the effects of Ferret VAChT-SAP treatment on retinal cell ablation, we performed a series of immunohistochemical labeling experiments on control and treated groups. Intraocular application of Ferret VAChT-SAP on P9 and P10 (see Materials and methods) leads to ablation of SACs by P25 as evidenced by a loss of ChAT(+) cell bodies in the ganglion cell layer and inner nuclear layer (Figure 1A-F) as well as a loss of Ferret VAChT antibody staining of SAC dendrites in the inner plexiform layer (Figure 1a, top right panel). Quantification reveals that Ferret VAChT-SAP treatment reduces SAC number from 27.93 ± 2.87 cells/mm in saline controls (mean ± standard error of the mean (SEM)) to 4.46 ± 0.99 cells/mm in Ferret VAChT-SAP-treated retinae (mean ± SEM), an 84% ablation (Figure 1b).

To evaluate the specificity of Ferret VAChT-SAP treatment, we immunolabeled retinae with antibody markers of different retinal neuron subtypes. We were particularly concerned about deleterious non-specific effects on RGCs and elected to use multiple markers of these cells to evaluate their status following Ferret VAChT-SAP treatment. Brn3a(+) and NeuN(+) RGCs were reduced in Ferret VAChT-SAP-treated retinae (26.96 ± 5.41 Brn3a(+) and 26.94 ± 1.48 NeuN(+) (mean cells/mm ± SEM)) relative to saline controls (35.07 ± 2.76 Brn3a(+) and 35.84 ± 2.62 NeuN(+) (mean cells/mm ± SEM)) (Brn3a, 23% ablation, *P *= 0.2060, Figure 1C,D; NeuN, 25% ablation, *P = 0.0447, Figure 1E,F).

**Figure 2:**
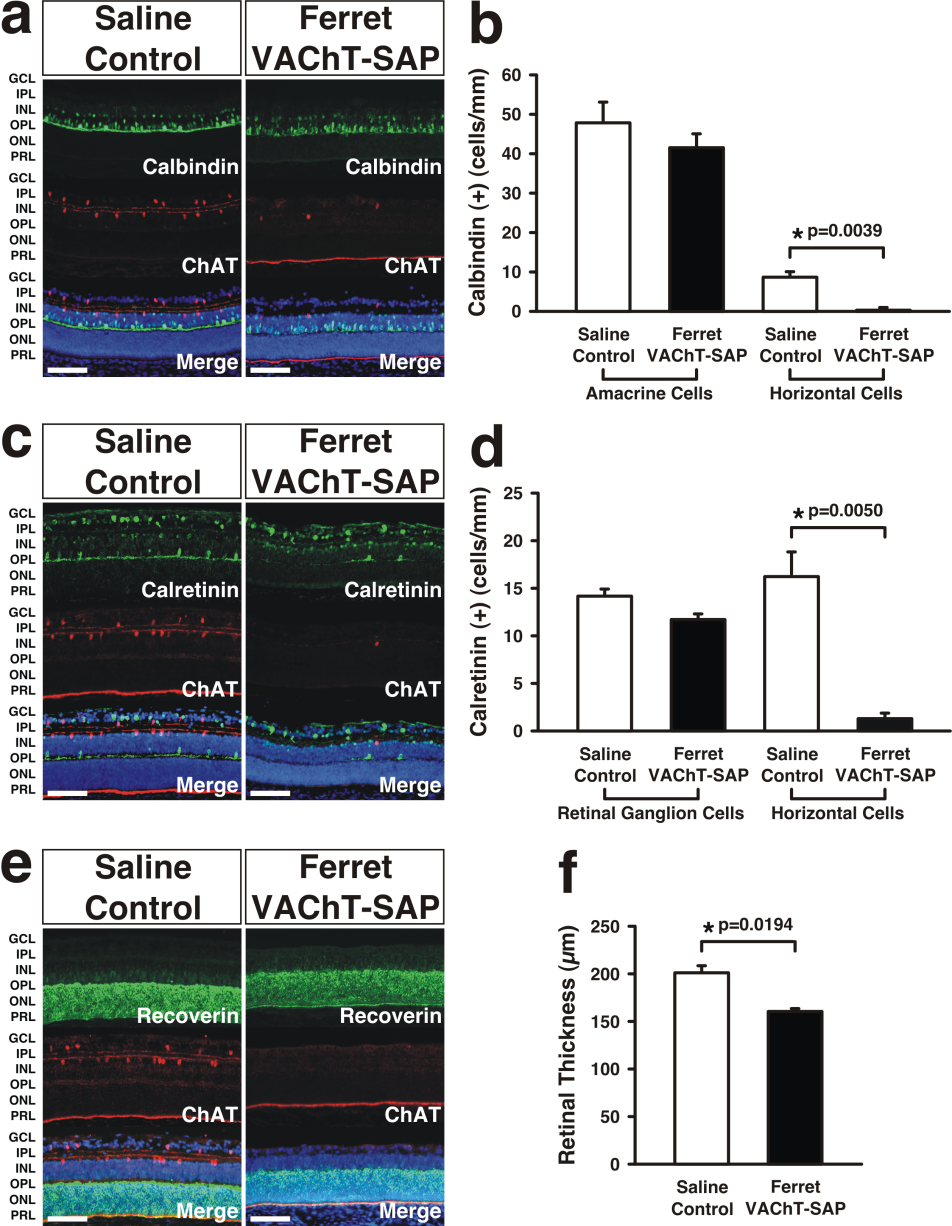
**Effects of Ferret VAChT-SAP treatment on other retinal neuron types**. **(A,B) **Treatment with Ferret VAChT-SAP leads to ablation of calbindin(+) amacrine cells and horizontal cells. **(C,D) **Calretinin(+) RGCs and HCs are also ablated by immunotoxin treatment. **(E,F) **Recoverin(+) photoreceptors appear qualitatively similar in both groups, but the retinal sheet is thinner in immunotoxin-treated retinae compared to controls. Data reflect means ± SEMs. Statistics reflect two-tailed *P*-values calculated from independent two sample *t*-tests. 'Merge' panels are counterstained with DAPI to reveal cytoarchitecture. GCL, ganglion cell layer; IPL, inner plexiform layer; INL, inner nuclear layer; OPL, outer plexiform layer; ONL, outer nuclear layer; PRL, photoreceptor layer. Scale bars: 100 μm.

In addition to ablating some RGCs, Ferret VAChT-SAP also resulted in the loss of horizontal cells (HCs) and some amacrine cells (ACs). Ferret VAChT-SAP treatment reduced calbindin(+) A-type HCs from 9.18 ± 1.31 cells/mm in saline controls (mean ± SEM) to 1.07 ± 0.48 cells/mm (mean ± SEM) in treated retinae, an 88% reduction (Figure 2A,B). Similarly, calbindin(+) ACs were reduced from 48.52 ± 4.99 cells/mm in saline controls (mean ± SEM) to 42.01 ± 3.44 cells/mm in Ferret VAChT-SAP-treated retinae (mean ± SEM) (Figure 2A,B). In saline control retinae, calretinin(+) A- and B-type HCs have a density of 16.46 ± 2.62 cells/mm (mean ± SEM) whereas treatment with Ferret VAChT-SAP ablated calretinin(+) HCs to a density of 1.5 ± 0.51 cells/mm (mean ± SEM), a 91% reduction (Figure 2C,D). Calretinin(+) RGC cell numbers were not significantly different between saline controls (14.34 ± 0.74 cells/mm ± SEM) and Ferret VAChT-SAP-treated retinae (11.88 ± 0.66 cells/mm ± SEM) (*P *= 0.0634; Figure 2C,D).

Recoverin labeling of rods, cones, and nascent bipolar cells in saline controls was qualitatively similar to that seen in Ferret VAChT-SAP-treated retinae, but quantification of the absolute thickness of the retina counterstained with DAPI (Figures 1 and 2A,C,E, merge panels) revealed a significant thinning of the retinal sheet in treated (162.31 ± 2.95 μm; mean ± SEM) versus control (203.32 ± 6.70 μm; mean ± SEM) groups (Figure 2F).

### Ferret VAChT-SAP disrupts spontaneous retinal activity

**Figure 3:**
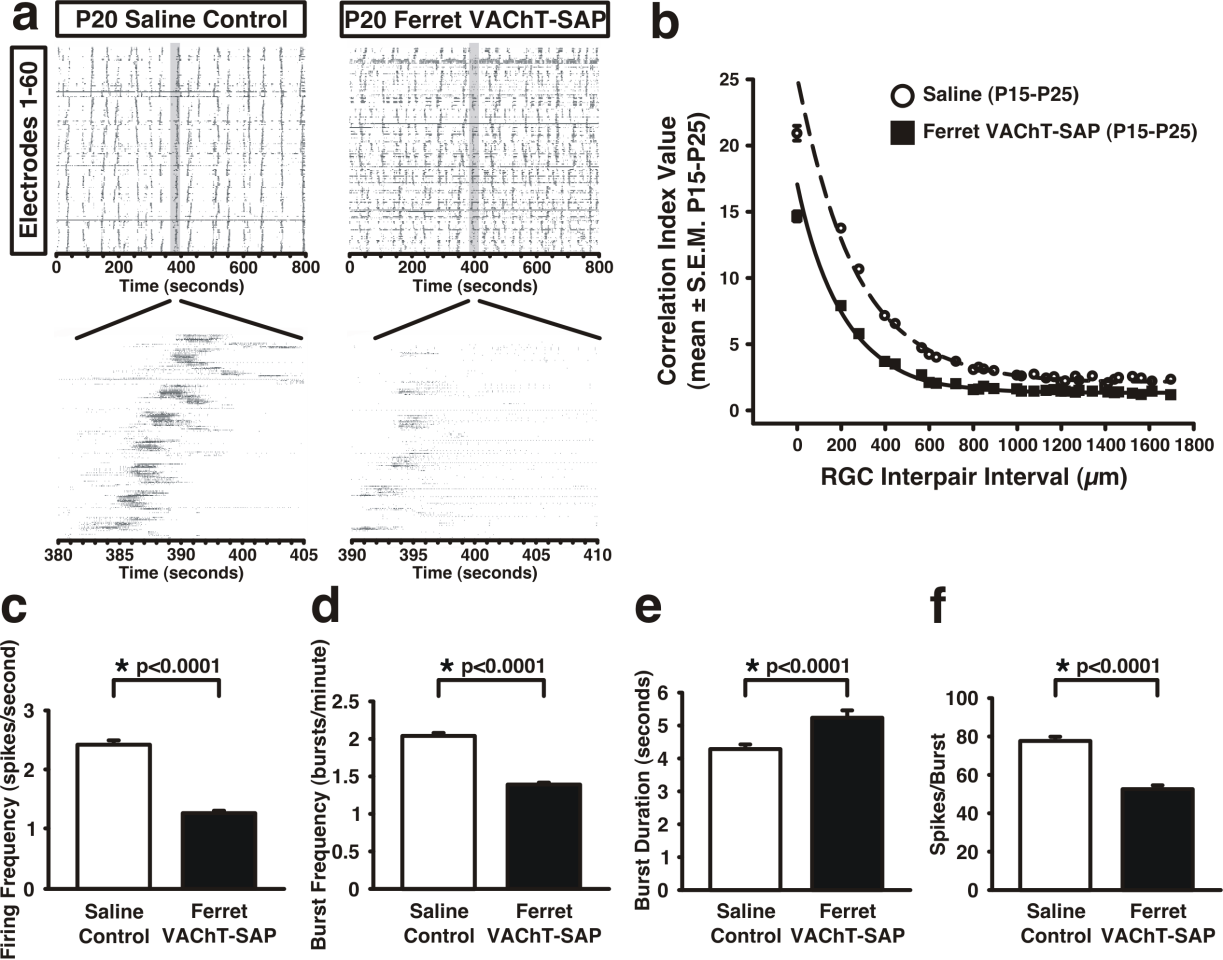
**Ferret VAChT-SAP treatment disrupts spontaneous retinal activity during P15 to P25**. **(A) **Raster plots from MEA recordings depict abnormal spontaneous retinal activity in Ferret VAChT-SAP-treated retinae compared to saline controls. **(B) **Correlation index analysis of RGC output shows that correlations are reduced at all inter-pair intervals in Ferret VAChT-SAP versus saline groups. **(C,D) **Ferret VAChT-SAP reduces overall activity levels (C) and overall RGC burst frequency (D) compared to saline controls. **(E,F) **Treatment with Ferret VAChT-SAP increases RGC burst duration (E) and decreases the number of spikes/burst (F). Panels (B-F) reflect means ± SEMs for grouped data from recordings at P15 (n = 2), P20 (n = 2), and P25 (n = 2). Statistics reflect two-tailed *P*-values resulting from independent two-sample *t*-tests.

To evaluate the effects of Ferret VAChT-SAP treatment on spontaneous retinal activity, we recorded spontaneous spiking from control and treated retinae using a multi-electrode recording array (MEA). Figure 3A depicts representative spike trains obtained by this technique from both control and Ferret VAChT-SAP-treated retinae from the same animal at P20. In controls, retinal waves swept across the patch of recorded retina several times each minute. Spreading waves (Figure 3A, left panel) lead to bursts of action potentials recorded on successive electrodes. Spike trains of neighboring RGCs in saline control recordings were highly correlated and correlations in spiking between RGC pairs dropped off as a function of inter-pair distance (Figure 3B, dashed line shows fit to the data; P15 to P25 grouped means ± SEMs). In contrast, Ferret VAChT-SAP-treated retinae exhibited significantly lower correlation coefficients at all RGC pair intervals (Figure 3b, solid line shows fit to the data; P15 to P25 grouped means ± SEMs). However, correlations between RGC pairs persisted in Ferret VAChT-SAP-treated retinae and propagating waves were evident (Figure 3A, right panels).

In addition to reducing the magnitude of correlations between RGC pairs, treatment with Ferret VAChT-SAP reduced the overall firing frequency of the ensemble of recorded RGCs. Firing frequency in saline control retinae was 2.49 ± 0.07 spikes/second and was reduced to 1.36 ± 0.04 spikes/second in Ferret VAChT-SAP-treated retinae (P15 to P25 grouped means ± SEMs; Figure 3C). Additionally, burst frequency was reduced from 2.06 ± 0.04 bursts/minute in saline control retinae to 1.41 ± 0.03 bursts/minute in Ferret VAChT-SAP-treated retinae (P15 to P25 grouped means ± SEMs; Figure 3D). Ferret VAChT-SAP reduced the total number of cells exhibiting bursts from 99.61% in saline controls to 96.59% in toxin-treated retinae (data not shown).

Treatment with Ferret VAChT-SAP also disrupted the structure of individual RGC spike trains. In saline control recordings, individual RGCs recruited into wavefronts exhibit long bursts of action potentials (4.34 ± 0.14 seconds, P15 to P25 grouped mean ± SEM; Figure 3E) with an average of 78.59 ± 1.98 spikes/burst (P15 to P25 grouped mean ± SEM; Figure 3F). In Ferret VAChT-SAP-treated retinae, bursts were significantly longer (5.28 ± 0.22 seconds, P15 to P25 grouped mean ± SEM; Figure 3E), but contained significantly fewer spikes on average than RGCs in control retinae (53.51 ± 1.71 spikes/burst, P15 to P25 grouped mean ± SEM; Figure 3F). The decrease in correlations between RGC pairs of Ferret VAChT-SAP-treated retinae (Figure 3B) was partly due to a significant increase in spike activity not confined to bursts. In saline control recordings 91.83 ± 0.40% of spikes occurred within bursts (P15 to P25 grouped mean ± SEM) while only 79.16 ± 0.80% of the total spikes recorded in Ferret VAChT-SAP-treated retinae were confined to bursts (P15 to P25 grouped mean ± SEM) (*P < 0.0001, independent two-sample *t*-test; data not shown). We also observed a small but significant decrease in the percentage of time RGCs fired at rates >10 Hz in Ferret VAChT-SAP-treated (1.71 ± 0.03%, P15 to P25 grouped mean ± SEM) versus saline control retinae (1.99 ± 0.02%, P15 to P25 grouped mean ± SEM) (*P < 0.0001, independent two-sample *t*-test; data not shown).

### Ferret VAChT-SAP treatment eliminates the intersublaminar space between On and Off sublaminae

**Figure 4:**
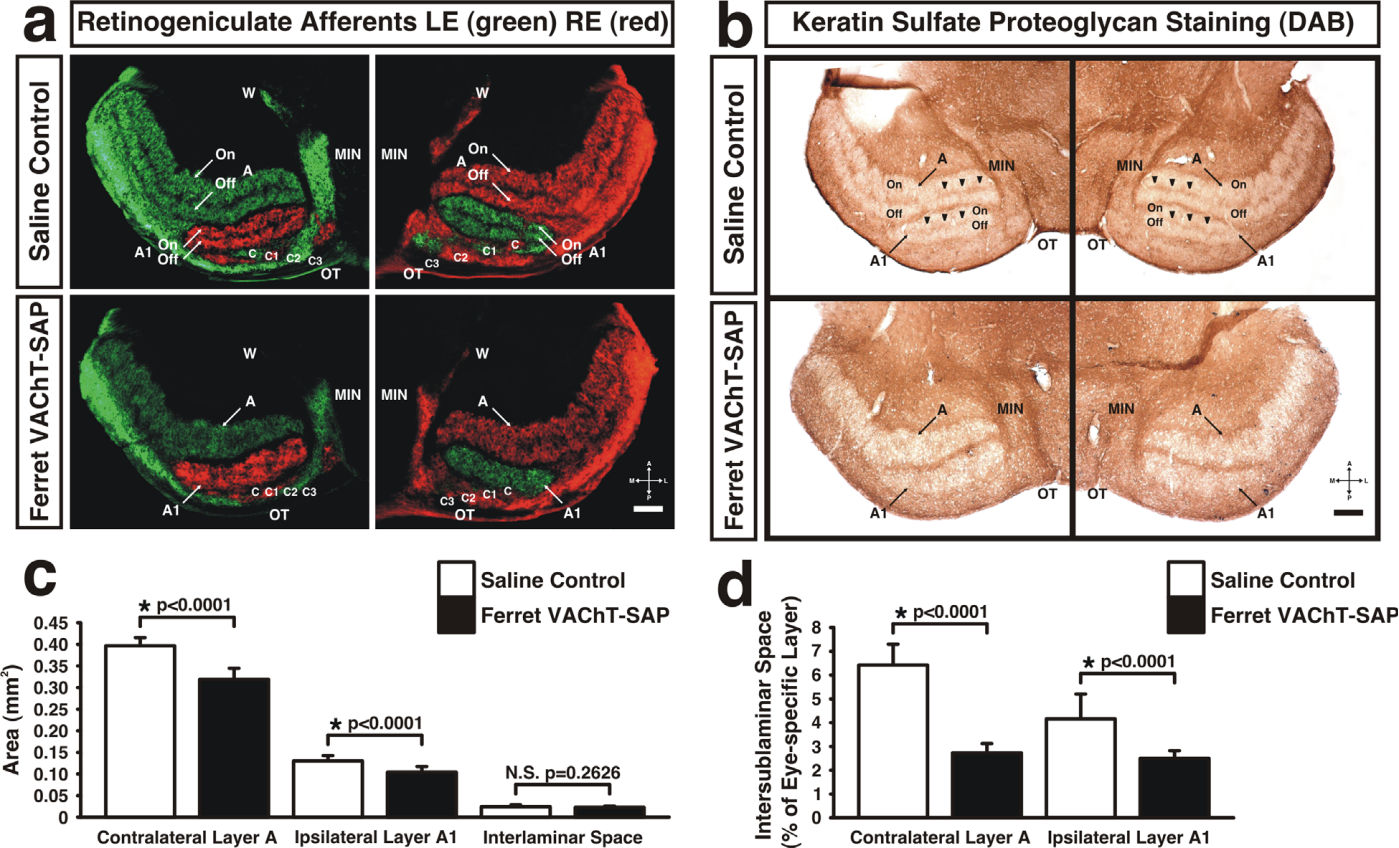
**Ferret VAChT-SAP disrupts development of On and Off sublamination**. **(A) **On and Off retinogeniculate afferents in both dLGNs (left and right panels) at P25 are separated by intersublaminar spaces into distinct sublaminae in saline controls (arrows in (A), upper panels), but sublaminae are absent in Ferret VAChT-SAP-treated ferrets (A, lower panels). **(A,C) **The size of contralateral and ipsilateral layers is reduced by Ferret VAChT-SAP treatment, but eye-specific segregation is normal and the interlaminar space is unaffected. **(B-D) **Treatment with Ferret VAChT-SAP prevents the normal expression of ABAKAN (indicated by arrowheads in upper panels of (B)) in the intersublaminar space between On and Off sublaminae, but not in the interlaminar space between eye-specific layers. A, major contralateral layer; A1, major ipsilateral layer; On, On-center sublamina; Off, Off-center sublamina; C, minor contralateral layer; C1, minor ipsilateral layer; C2, minor contralateral layer; C3, retinal-afferent-free layer; W, geniculate wing; MIN, medial intralaminar nucleus. Panels (C,D) reflect means ± SEMs; statistics reflect two-tailed *P*-values resulting from independent two-sample *t*-tests. Coordinate insets in (A,B) reflect anterior-posterior and medial-lateral orientation within the dorsal thalamus. Scale bars: 200 μm. N.S., not significant.

Anterograde retinogeniculate labeling in saline control ferrets at P25 reveals a retinal-afferent-free zone splitting each eye-specific lamina into On and Off sublaminae (Figure 4A). In ferrets treated with Ferret VAChT-SAP at P9/P10, retinogeniculate projections from the two eyes at P25 form contralateral (A) and ipsilateral (A1) eye-specific layers with no margin of separation between On and Off sublaminae (Figure 4A). Treatment with Ferret VAChT-SAP reduces the area of the contralateral (Figure 3B; 0.321 ± 0.026 mm^2 ^in Ferret VAChT-SAP; 0.399 ± 0.019 mm^2 ^in saline controls; means ± SEMs) and ipsilateral eye-specific layers (Figure 4C; 0.107 ± 0.013 mm^2 ^in Ferret VAChT-SAP; 0.132 ± 0.012 mm^2 ^in saline controls; means ± SEMs), but has no effect on the maintenance of segregation between afferents of the two eyes (Figure 4A, quantification not shown). In our quantification of the absolute area of each eye-specific layer in saline controls, we were careful to exclude the intersublaminar space to provide an accurate comparison to the areas measured in Ferret VAChT-SAP-treated cases in which these spaces were absent (see Materials and methods). The reduced size of eye-specific layers in Ferret VAChT-SAP-treated animals is consistent with our finding that some RGCs are ablated in immunotoxin-treated retinae (Figure 1).

In controls at P25, an astrocyte-associated keratin sulfate proteoglycan, ABAKAN, is expressed in the interlaminar space between eye-specific layers and in intersublaminar spaces between On and Off sublaminae within each eye-specific layer of the dLGN (Figure 4B, arrowheads in upper panels). Treatment with Ferret VAChT-SAP prevents the normal expression of ABAKAN between On and Off sublaminae (Figure 4B, lower panels). The expression of ABAKAN within each eye-specific layer is significantly reduced in Ferret VAChT-SAP-treated animals (Figure 4D; 2.79 ± 0.39% of contralateral layer A; 2.55 ± 0.33% of ipsilateral layer A1; means ± SEMs) compared to controls (Figure 4D; 6.45 -± 0.89% of contralateral layer A; 4.21 ± 1.04% of ipsilateral layer A1; means ± SEMs). The lack of staining within each eye-specific layer seen in Ferret VAChT-SAP-treated cases is not due to a defect in our immunolabeling protocol as the expression of ABAKAN between eye-specific layers (interlaminar space) was normal in both Ferret VAChT-SAP and saline control groups in every case (Figure 4C; 0.026 ± 0.002 mm^2 ^in Ferret VAChT-SAP; 0.027 ± 0.004 mm^2 ^in saline controls; means ± SEMs). We were careful to investigate the quality of ABAKAN labeling at multiple levels of the dLGN in both controls and Ferret VAChT-SAP-treated groups and noted that small patches of ABAKAN label were occasionally present in the dLGNs of Ferret VAChT-SAP-treated animals. These patches were quantitatively measured in our analysis of ABAKAN staining patterns (see Materials and methods) and account for the non-zero area of the intersublaminar spaces we measured in Ferret VAChT-SAP-treated animals (Figure 4D). Importantly, we never encountered an extended leaflet of ABAKAN label corresponding to the intersublaminar space in any of the Ferret VAChT-SAP-treated cases.

### The intersublaminar space is not necessary for functional On and Off segregation in the dLGN

Our anatomical results indicate a loss of On and Off sublaminar segregation as evidenced by the lack of pre- and post-synaptic intersublaminar spaces within each eye-specific layer of the dLGN. In order to evaluate the effects of eliminating the intersublaminar spaces on the functional organization of On and Off pathways, we performed single-unit electrophysiological recordings in the dLGNs of animals treated with either Ferret VAChT-SAP or saline at P9/P10 and allowed to recover into adulthood (>P100).

**Figure 5:**
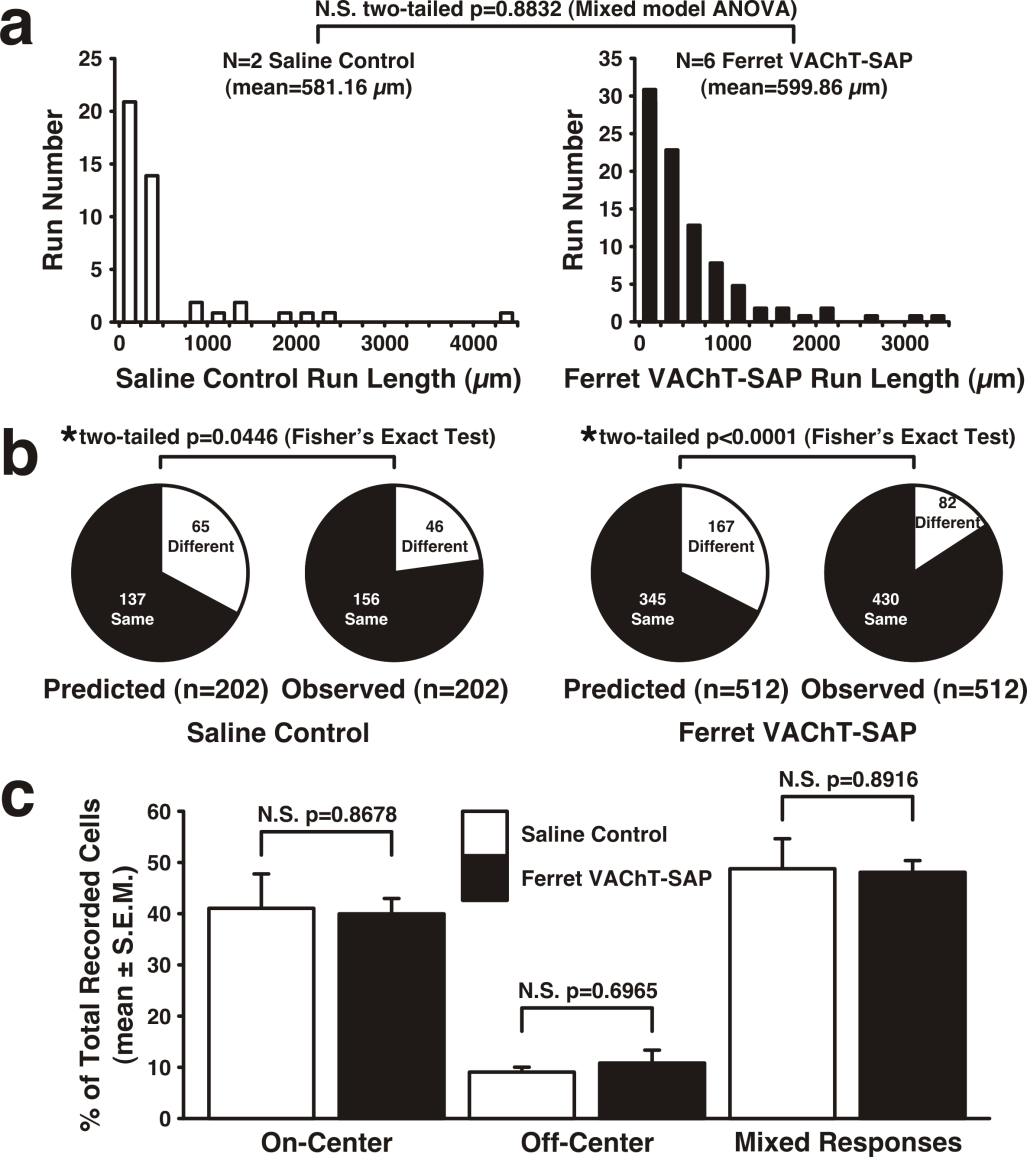
**Ferret VAChT-SAP treatment does not disrupt physiological On and Off organization in the dLGN**. On and Off responsive units are located along runs of same center-type response in both saline and Ferret VAChT-SAP recovery animals. **(A) **Distributions of same center-type run lengths are not significantly different between the groups. **(B) **Nearest neighbor analysis of On and Off organization reveals that, for both saline and Ferret VAChT-SAP groups, On and Off units exhibit significantly greater clustering with neighbors of the same center-type than would be predicted by a randomized distribution of the cell populations. **(C) **The proportions of On/Off/Mixed responses recorded in the dLGNs of saline and Ferret VAChT-SAP cohorts are not different for any of the response types. Statistics in (C) reflect two-tailed *P*-values resulting from independent two-sample *t*-tests. N.S., not significant.

Upon entering the dLGN in all recordings, On and Off responsive units were often found intermingled or in small clusters that periodically alternated, an arrangement consistent with the known physiological organization of the C laminae in the ferret [1]. As we continued along each penetration in saline control recordings we observed runs of consecutive On or Off-center responsive units in both the contralateral and ipsilateral layers of the dLGN with an average run length of 581.16 ± 559.60 μm (mean ± SEM) (Figure 5A). In animals treated with Ferret VAChT-SAP, similar runs of On- and Off-center activity were recorded in each eye-specific layer of the dLGN with an average run length of 599.86 ± 259.71 (mean ± SEM; Figure 5a). In both saline and Ferret VAChT-SAP groups, the center-sign (On or Off) of same center-type runs was often observed to switch as the penetration progressed, indicative of the electrode crossing from one sublamina into the next. We mapped the dLGNs of six Ferret VAChT-SAP recovery animals and in every case we encountered long runs of same center-type responses. Statistical comparison of run length distributions recorded in saline and Ferret VAChT-SAP groups revealed no significant difference between the groups (Figure 5A; *P *= 0.8832 mixed model ANOVA).

To further assess the organization of On and Off responses in the dLGN, we performed an analysis of nearest neighbor relations amongst identified On and Off cells along each penetration (see Materials and methods). Along each penetration, each identified On or Off cell has nearest neighboring cells with response types either the same or different from that particular cell. If On or Off cells tend to be clustered in laminae, the probability of identifying neighbors of the 'same-center type' should be greater than that predicted by a binomial model that randomizes the order of the On and Off units recorded (see Materials and methods). In our sample of 202 saline control neurons of a single-center type (161 On-center and 41 Off-center), 137 should have neighbors of the same center-type if the organization of On and Off responses is random in the dLGN (Figure 5B). We observed that 156 out of 202 cells had neighbors of the same center-type, a fraction significantly greater than that predicted by the binomial model (Figure 5B; *P = 0.0446, Fisher's exact test). This would be the case if On and Off cells exhibit greater same center-type clustering than that predicted by a random arrangement of the observed cells. This result is in agreement with the known physiological organization of On and Off sublaminae in the ferret dLGN [1]. In our Ferret VAChT-SAP-treated recordings, we identified 407 On-center cells and 105 Off-center cells, leading us to predict that 345 cells would have neighbors of the same center-type if the cells were randomly distributed within the dLGN (Figure 5B). We observed that 430 recorded cells had neighbors of the same-center type, a value reflective of significantly greater On and Off clustering than that predicted by the randomization model (Figure 5B; *P < 0.0001, Fisher's exact test).

To evaluate whether treatment with Ferret VAChT-SAP altered the overall proportions of On and Off responses recorded in the dLGN, we compared the relative fractions of the total population for each response type. For the total population of saline control multi-unit recordings, 41.40% of recording sites were strictly On-center-type and 9.47% were Off-center-type (Figure 5C). The remaining 49.13% were of Mixed center-type, which reflects responses in both the C-laminae and along the borders of On and Off sublaminae within each eye-specific layer (Figure 5C) [1]. In multi-unit recordings from Ferret VAChT-SAP-treated ferrets, 40.30% of recording sites had only On-center responses, 11.28% had only Off-center responses, and 48.42% had Mixed center-type responses, values not significantly different from saline controls (*P *= 0.8678 (On), *P *= 0.6965 (Off), *P *= 0.8916 (Mixed); two-sample independent *t*-test two-tailed *P*-values; Figure 5C).

**Figure 6:**
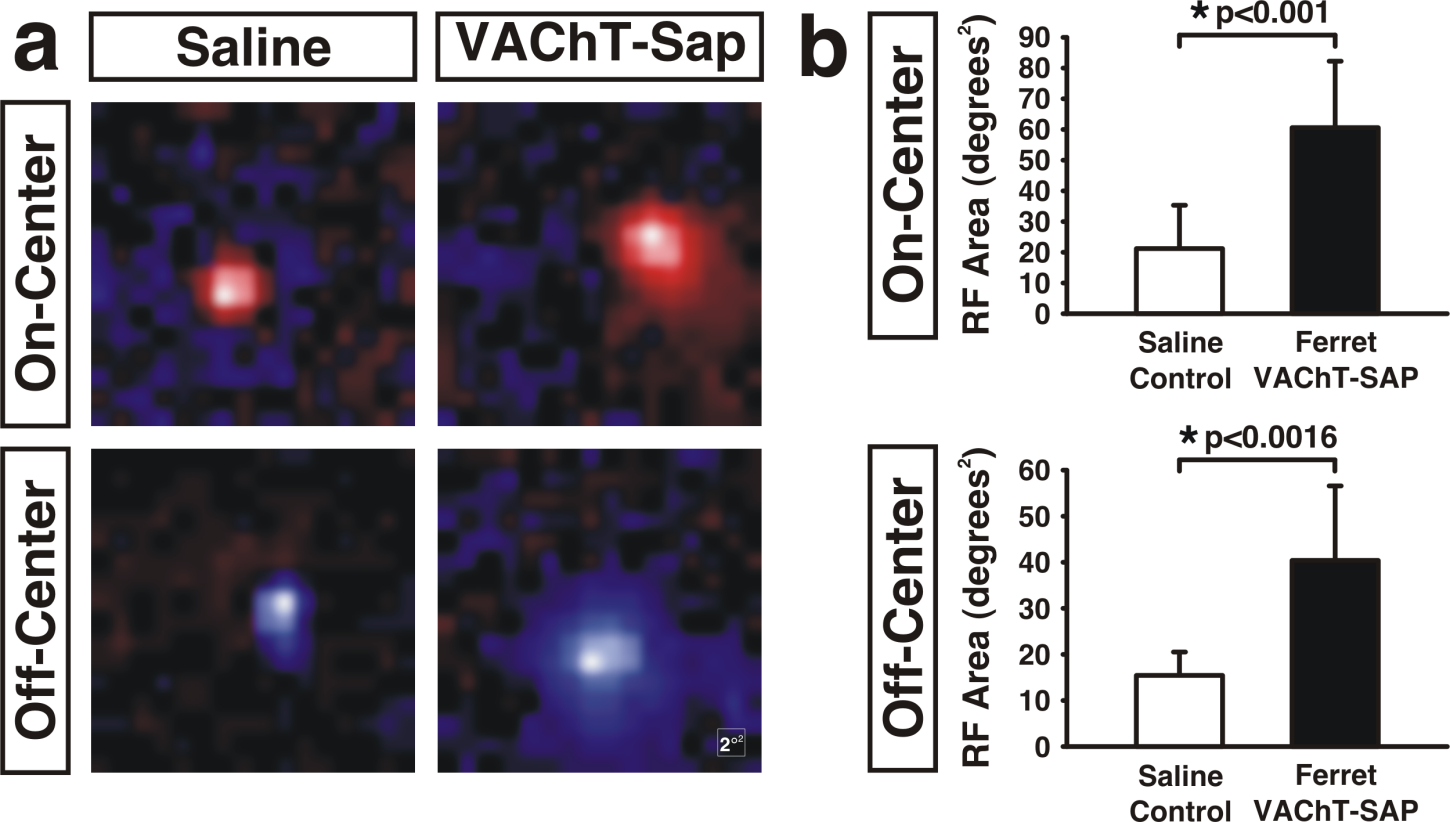
**Ferret VAChT-SAP treatment disrupts receptive field size of On- and Off-center dLGN neurons**. **(A) **On and Off responsive units are center-surround organized and of a single center-type, either On-center or Off-center in both saline and Ferret VAChT-SAP-treated groups (scale inset equals 2°^2 ^of visual angle). **(A,B) **Treatment with Ferret VAChT-SAP leads to enlarged receptive fields for both On-center and Off-center neurons recorded in the dLGN (means ± SEMs). Statistics reflect two-tailed *P*-values calculated from independent two sample *t*-tests.

In addition to evaluating the clustering and frequency of On and Off responses recorded in the dLGN, we also quantitatively mapped the receptive field (RF) structure of individual cells along our electrode penetrations. RFs in saline control ferrets were center-surround organized and of a single center-type response (either On or Off; Figure 6A, left panels). Offline analysis of spike trains recorded at sites of mixed responsiveness revealed the presence of multiple cells exhibiting either On- or Off-center-type RFs, but never RFs with dual On-center and Off-center subfields. This is consistent with Mixed responses being reflective of signals picked up from the borders of closely apposed On and Off regions such as those found in the C-laminae and along the intersublaminar regions of each eye-specific layer [1]. For neurons recorded in Ferret VAChT-SAP recovery animals, RFs were also center-surround organized with a single center-type, either On-center or Off-center, and never with dual On-center and Off-center regions in the same RF (Figure 6A, right panels). However, the RF area of On-center cells in saline controls was 21.68 ± 13.85°^2^, but was expanded to 61.07 ± 21.4°^2 ^in Ferret VAChT-SAP-treated animals (Figure 6B, upper panel; means ± SEMs). Similarly, Off-center cells in saline controls had RFs measuring 15.75 ± 4.91°^2 ^but were expanded to 40.69 ± 16.05°^2 ^in Ferret VAChT-SAP-treated animals (Figure 6B, lower panel; means ± SEMs).

## Discussion

Disrupting spontaneous activity in the retinogeniculate pathway perturbs the anatomical development of segregated On and Off sublaminae in the ferret dLGN, but the physiological relevance of such defects is unknown. Likewise, glial boundaries define the border between On and Off sublaminae in the developing ferret dLGN, but a role for glia in sublaminar refinement has not been demonstrated. In this study we disrupted retinal circuitry during the development of On and Off sublaminae by ablating several retinal cell types with an immunotoxin. This treatment altered spontaneous retinal activity and disrupted the anatomical hallmarks of On and Off segregation, including expression of a glial-derived repulsive signaling molecule, ABAKAN, hypothesized to contribute to the developmental segregation of On and Off sublaminae. In contrast to predictions based on previous work, the activity-dependent anatomical disruption of segregated On and Off sublaminae induced by immunotoxin treatment did not affect the functional organization of On and Off channels in the dLGN. These results demonstrate that intersublaminar spaces and inhibitory barriers of ABAKAN expression are dispensable for the normal segregation of functional On and Off channels in the ferret retinogeniculate pathway.

### Contributions of spontaneous and visually driven activity to sublaminar segregation

Disruption of spontaneous activity in the retinogeniculate pathway is known to prevent the emergence of anatomically discrete On and Off sublaminae [10-12]. Our results support these previous findings by showing that abnormal spontaneous retinal activity induced by Ferret VAChT-SAP treatment correlates with a lack of discrete segregation of On and Off sublaminae within each eye-specific layer. In an advance from previous studies, our results demonstrate the abolishment of intersublaminar spaces as seen via both anterograde dye labeling of retinogeniculate afferents as well as by loss of ABAKAN expression between On and Off territories in the dLGN. Remarkably, our data demonstrate that eliminating intersublaminar spaces does not affect the physiological arrangement of On and Off laminar channels in the ferret dLGN. Previous work from our laboratory showed that both retinotopy and the receptive field organization of individual relay neurons in the dLGN are normal following activity manipulations that abolish the stereotypical pattern of eye-specific laminar and On and Off sublaminar development in the ferret [14]. Thus, manipulations of spontaneous retinal activity that distort the gross anatomical pattern of retinogeniculate inputs seen in normal ferrets do not inevitably disrupt all physiological features of the system. Although the dissociation of anatomical sublamination and physiological function reported here is perhaps surprising, it is not without parallel. Rodents lack distinct cytoarchitectural laminae in the dLGN [15,16] and yet retinal afferents are anatomically segregated from one another [17,18] to form distinct physiological channels in the retinogeniculate pathway [19-21]. In light of this species difference, our present findings highlight the outstanding question of the function, if any, for overt lamination in the ferret dLGN.

In our single-unit recordings in the dLGNs of both saline and Ferret VAChT-SAP-treated ferrets, receptive fields of On and Off cells were always of a single center-type and did not display receptive field discontinuities normally seen in juvenile ferrets [22]. This is not surprising considering the animals in our study experienced normal vision, initially through the closed eyelids and then again for >100 days after eye-opening. It is possible that visual input, known to drive strong correlations in spiking amongst same center-type cells of the dLGN [23,24], may be sufficient to remediate receptive fields that were perhaps initially abnormal due to immunotoxin treatment.

In support of a role for visual experience in On and Off segregation, blockade of retinal activity after eye-opening prevents the maturation of retinogeniculate synapses [25] and leads to receptive fields with discrete, spatially discontinuous On and Off regions responsive to light increments and decrements, respectively [26,27]. Similarly, dark-rearing leads to abnormal receptive fields with discrete On and Off 'hot spots' that underlie single dLGN neurons' responsiveness to light and dark stimuli [24].

Although we did not observe center-sign defects in RFs of On- or Off-center units in our experiments, we did observe that RFs of both On- and Off-center cells were significantly larger in Ferret VAChT-SAP-treated ferrets than in saline controls. This likely reflects non-specific damage to the lens induced by the immunotoxin, but we cannot rule out defects in the refinement of retinal and/or retinogeniculate circuitry due to Ferret VAChT-SAP treatment. Abnormal retinal activity induced genetically, pharmacologically, or by dark-rearing is known to disrupt the development of retinal circuitry [28-37] as well as refinement of On and Off retinogeniculate projections [10,24,26,27] and could lead to expanded RFs of RGCs and/or dLGN neurons such as those we observed.

Importantly, the disruption of spontaneous retinal activity induced by Ferret VAChT-SAP did not affect eye-specific segregation or the expression of ABAKAN between eye-specific layers. This is in contrast to reports that altering or silencing spontaneous retinal activity can impact the maintenance of segregated eye-specific layers following their initial segregation [38,39]. In the future it will be interesting to investigate the expression pattern of ABAKAN in ferrets where the maintenance of eye-specific segregation is disrupted [38]. Our results are in agreement with other studies showing that disruption of spontaneous activity in the retinogeniculate pathway during the period of On and Off segregation does not impact the maintenance of segregated eye-specific layers [10-12]. In animals treated with Ferret VAChT-SAP, it may be the case that features of activity necessary for the maintenance of eye-specific segregation remain intact while those necessary for intersublaminar space development are disrupted. In our recordings, correlations between RGC pairs, burst structure of individual RGCs, and overall activity levels were each affected by the immunotoxin treatment and it is impossible to determine which properties of spontaneous retinal activity are responsible for the observed defects in On and Off anatomical sublamination. The features of spontaneous retinal activity critical for maintaining segregated eye-specific layers versus those critical for the emergence of segregated On and Off sublaminae remain unknown.

Additionally, it is possible that phenotypes we observed may result from altered molecular expression in RGC axons resulting from effects of the immunotoxin on retinal circuitry. However, the anatomical abnormalities we observed in the dLGNs of immunotoxin-treated ferrets are remarkably similar to those seen following pharmacological manipulations of spontaneous retinal activity [10-12], consistent with a role for disrupted patterns of spontaneous retinal activity in the abnormal anatomical segregation of On and Off sublaminae seen in the dLGNs of immunotoxin treated ferrets.

It has been argued that differential firing patterns between On and Off RGCs may drive the segregation of On and Off retinogeniculate channels via Hebbian mechanisms [40-43]. However, a direct role for the observed firing patterns in the establishment of segregated On and Off sublaminae has not been established. In an effort to address this issue, we attempted to record the different patterns of activity exhibited by On and Off responsive RGCs during spontaneous retinal waves in control and Ferret VAChT-SAP-treated retinae. Unfortunately, we were unable to drive visual responses in either On-center or Off-center RGCs of the ferret retina between P15 and P25 *in vitro *and thus could not use physiological methods to individuate the different patterns of activity in these two broad types of RGC during spontaneous retinal activity in our MEA recordings (data not shown).

### A role for glia in the developmental refinement of retinogeniculate projections

ABAKAN, an astrocyte-associated repulsive cue [4,8,9,44], has been hypothesized to act as a barrier between emerging On and Off sublaminae by constraining ingrowing retinal afferents and/or neurites of developing cells in the dLGN [4]. Consistent with this proposal are results showing that individual retinogeniculate axons have abnormally positioned terminals within each eye-specific lamina in animals lacking intersublaminar spaces due to retinal activity manipulations [11,12]. Our finding that disruption of spontaneous retinal activity eliminates the expression of ABAKAN between developing On and Off sublaminae without a concomitant change in the functional segregation and organization of On and Off domains suggests that ABAKAN is not necessary for the normal development of these functional channels. ABAKAN may still be important for constraining the extent of On and Off retinogeniculate afferents and our data do not rule out a role for ABAKAN in the precise positioning of individual retinogeniculate terminals within each eye-specific layer. However, the physiological segregation and same center-type clustering of On and Off responses we recorded in the dLGNs of Ferret VAChT-SAP-treated animals indicate considerable precision in the refinement of synaptic specificity between On and Off RGCs and target neurons in the dLGN. This is perhaps not surprising in light of studies showing that X-type RGCs, which constitute the bulk of On and Off specificity [45], are less susceptible to perturbations of retinal activity than Y-type RGCs [46-52]. Thus, the anatomical defects we observed in the current study may reflect defects of Y-cell projections to a greater extent than X-cell counterparts.

While our data show that disrupting ABAKAN expression did not affect functional segregation of On and Off channels, it is possible that other molecular cues expressed between On and Off subdomains [7] were unaffected by Ferret VAChT-SAP treatment and were sufficient to direct the segregation of On and Off retinogeniculate inputs. The cellular and molecular compositions of the interlaminar and intersublaminar margins remain largely unexplored.

## Conclusions

The results of our study demonstrate that functional organization of On and Off channels in the ferret dLGN is not impacted by manipulations of spontaneous retinal activity that disrupt anatomical hallmarks of On and Off sublamination. Additionally, we have demonstrated that disrupted expression of a repulsive signaling molecule hypothesized to contribute to the developmental segregation of On and Off sublaminae did not affect the emergence of these functional channels. These results highlight an important developmental dissociation between anatomical and physiological sublamination in the On and Off pathways and call into question the significance of anatomical sublaminae in the ferret visual system.

## Materials and methods

### Animals

Timed-pregnant ferret jills were obtained from Marshall Farms (New Rose, NY, USA). P0 was defined as the day of birth. All experimental procedures were performed in accordance with the National Institutes of Health guidelines under protocols approved by the Animal Care and Use Committee of the University of California, Davis.

### Immunotoxin generation

The coding region for the carboxy-terminal domain of the VAChT protein was cloned from a ferret cDNA library using standard molecular techniques. The primers used were: sense primer 5'-TACGCGCTCGGGCCCATAGT-3'; antisense primer 5'-TGGAGGAGAAGCGGGTCTGCT-3'.

PCR products were sequenced and suitable antigenic regions were determined. Rabbits were immunized with a synthetic 20 amino acid peptide (sequence: RRSRSERDVLLDEPPQGLYD) corresponding to an epitope encoded in the carboxy-terminal region of our cloned *VAChT *cDNA sequence (Open Biosystems, Huntsville, AL, USA). Whole-animal serum was affinity-purified and shipped to our laboratory for verification of antibody specificity (see 'Retinal immunohistochemistry' section). SAC-specific affinity-purified ferret VAChT antibody was conjugated to Saporin toxin (Advanced Targeting Systems, San Diego, CA, USA).

### Immunotoxin treatment

The injection protocol was adapted from [3]. P9 ferrets were anaesthetized with inhalant isoflurane and 2 μl of Ferret VAChT-SAP (0.88 μg/μl) was injected. This protocol was repeated on P10. Control eyes were injected with equivalent volumes of 0.9% sterile saline. Following injections, the injection needle was allowed to remain stably inserted into the eye for a period of 3 minutes to allow for diffusion of the injection within the vitreal fluid of the eye. Animals for MEA recording were injected with Ferret VAChT-SAP in the left eye and saline in the right eye, thereby providing within-animal controls. Animals for anatomical and physiological study of retinogeniculate development were injected binocularly with either Ferret VAChT-SAP or saline of equivalent volumes in the two eyes.

### Surgery and tissue preparation for MEA recording

The procedures for retinal dissection for MEA recording have been described previously [53]. Neonatal ferrets were administered a lethal dose of Euthana-6 (0.1 ml, pentobarbital sodium; Western Medical Supply, Arcadia, CA, USA). Animals were enucleated and the retinae were removed and maintained in buffered, oxygenated media (Eagle's minimum essential medium (MEME), Sigma-Aldrich, St Louis, MO, USA) at room temperature. Sections of retinae measuring approximately 5 to 8 mm^2 ^were cut for recording on the MEA.

### Multielectrode array recordings

Retinal sections were placed ganglion cell layer down onto a 60-channel MEA (Multi-Channel Systems, Tubingen, Germany), held fixed by a dialysis membrane (Spectrapore 132130; Spectrum, Los Angeles, CA, USA), and superfused with buffered/oxygenated medium (MEME; Sigma-Aldrich) at 1 to 2 ml/minute at 37°C. Array electrodes were 30 μm in diameter, arranged on an 8 × 8 rectilinear grid with 200-μm interelectrode spacing. At this interelectrode spacing, the signal of a given cell appeared on only one electrode, so each cell was assigned the coordinates of the electrode that recorded its signal. Analog data were acquired at 20 kHz per channel simultaneously from each of the 60 electrodes. Following experimental setup, retinae were allowed to acclimate for 5 to 20 minutes on the MEA. On emergence of retinal wave epochs, recordings were performed for a period of 15 to 20 minutes, during which time overall firing rates of the ensemble appeared stable. Ferret-VAChT-SAP-treated retinae (P15, n = 2; P20, n = 2; P25, n = 2) were recorded first, after which the MEA was cleaned. Control saline treated retinae (P15, n = 2; P20, n = 2; P25, n = 2) were then prepared identically.

### Spike identification

Spike sorting and cell identification were performed identically to that in [53]. Before sorting spike events, the data were digitally filtered with a 125-Hz high-pass filter (four-pole Butterworth). A threshold of 6SD was set for each channel and 1 ms of data before a threshold-crossing event and 4 ms after the threshold event were stored for each negative-slope event. These candidate spike waveforms were then sorted with the OfflineSorter (Plexon, Denton, TX, USA) using the first three principal components of the spike waveforms. Coincident events within 0.5 ms of one another that occurred on all electrodes were attributed to perfusion noise and removed. Clusters were first identified using an EM (expectation-maximization) cluster algorithm by [54] and then manually edited for clustering errors. Typically, each electrode recorded the activity of one to three cells.

### Statistical analysis

#### Burst analysis

Burst analysis was modified from [53]. The burst duration was measured using the burst analysis algorithm provided by Neuroexplorer (Nex Technologies, Littleton, MA, USA). The spike train was scanned until an interspike interval (ISI) of ≤0.1 s was found. This defined the beginning of the burst. Subsequent spikes with ISIs of <1 s were included in the burst, whereas an ISI of >1 s denoted the end of the burst. If an interval between two bursts was <5 s, the two bursts were merged and considered to be one burst. Bursts with a duration <0.5 s or with fewer than four spikes were discarded. Isolated cells exhibiting fewer than five bursts during the recording were excluded from the burst analysis. Spike trains from cells excluded in the burst analysis were included in the analysis of overall retinal activity as well as in the measure of percentage of cells exhibiting bursts. The burst analysis algorithm provided the burst frequency, burst duration, interburst interval, ISI of spikes within bursts, number of spikes per burst, and percentage of spikes in bursts relative to total spike number for the recording. Two-sample independent *t*-tests were applied to the data for group comparison.

#### Correlation analysis

Correlation analysis was identical to that in [53]. To quantify the degree of correlated firing between recorded pairs of cells, all cross-correlation functions were calculated and assigned a correlation index (CI). The CI measures the likelihood relative to chance that a pair of cells fired together within a particular time window. The CI was computed as described by [55] using the following formula:

where N_ab_(-w, + w) is the number of spike pairs from cells a and b for which cell b fires within w seconds of cell a, T is the duration of the recording in seconds, N_a_(0, T) and N_b_(0, T) are the total number of spikes from cell a and b during the recording, and 2 × w is the width of the correlation window. N_ab _was computed using w = 0.1 s and the cross-correlation function was binned at 0.05 s. The particular values of the CI depend on the choice of the correlation window w. A value of 0.1 s was chosen based on what has been commonly used by other investigators as a reasonable timescale for activity-dependent modification of synaptic strength [56].

### Retinal immunohistochemistry

Following MEA recordings, dissected retinae and recorded retinal pieces were collected and fixed briefly (10 minutes) in 4% paraformaldehyde (Sigma-Aldrich) in 0.1 M phosphate buffer. Retinae were then rinsed several times in 0.1 M phosphate buffer and processed for whole-mount immunohistochemistry to evaluate SAC ablation. For whole mount, relieving cuts were made to allow the retina to lay flat. Retinae were incubated in blocking solution (10% donkey serum, 0.3% Triton-X 100 in 0.1 M phosphate buffer) for 4 h, then transferred to primary antibodies (1:50 goat anti-ChAT, Millipore, Billerica, MA, USA); 1:100 mouse NeuN, Millipore) for 48 h at 4°C, washed in 0.1 M phosphate buffer overnight, transferred to secondary antibody (1:200 donkey anti-goat Alexa 488, 1:200 donkey anti-mouse Alexa 594; Invitrogen, Carlsbad, CA, USA) for 2 h at room temperature, washed in 0.1 M phosphate buffer 5 × 30 minutes, mounted, and cover-slipped with Vectashield (Vectorlabs, Burlingame, CA, USA).

For thin-section retinal immunohistochemistry, retinae were immersed in 30% sucrose, cryosectioned at 18 μm, and mounted on positive coated slides. Sections were incubated in blocking solution (10% donkey serum, 0.3% Triton-X 100 in 0.1 M phosphate buffer) for 4 h, transferred to primary antibodies (1:50 goat anti-ChAT, Millipore; 1:100 mouse anti-NeuN, Millipore; 1:1,000 mouse anti-Calbindin, Sigma; 1:500 rabbit anti-Calretinin, Millipore; 1:500 rabbit anti-VAChT, custom antibody (see 'Immunotoxin generation' section); 1:50 Brn3a, Millipore; 1:1,000 rabbit anti-Recoverin; Millipore) overnight at 4°C, washed 5 × 30 minutes and then again overnight in 0.1 M phosphate buffer, transferred to secondary antibodies (1:200 donkey anti-mouse Alexa 594, 1:200 donkey anti-rabbit Alexa 488, 1:200 donkey anti-goat Alexa 488 or 594; Molecular Probes) for 2 h at room temperature, washed in 0.1 M phosphate buffer 5 × 30 minutes, and cover-slipped with Vectashield plus DAPI (Vectorlabs).

Immunostained retinae were imaged and 1-mm sections of retina were cropped at a distance of 500 μm on either side of the optic nerve head. Two cropped fields from each imaged retinal section were acquired and four to six sections per retina were imaged for each retinal label analyzed. Individual cells within each image were counted using the Cell Counter PlugIn in ImageJ [57]. N = 4 saline control retinae; N = 3 Ferret VAChT-SAP-treated retinae.

### Retinogeniculate labeling and analysis

Anterograde dye-tracing of retinogeniculate afferents was performed as in [3]. Sections were cut at 40 μm in the horizontal plane of section through the center of the dLGN. In each animal, five to seven sections from each side of the dorsal thalamus were thresholded at 30% above background as in [3]. Thresholded images were quantified in ImageJ [57]. Area measurements of contralateral and ipsilateral eye-specific layers were independently made after subtraction of the intersublaminar spaces. N = 3 saline controls (6 dLGNs); N = 5 Ferret VAChT-SAP animals (10 dLGNs).

### ABAKAN immunohistochemistry and analysis

All anti-ABAKAN immunohistochemistry was performed at P25 as this molecule is expressed transiently in the intersublaminar spaces during the period of On/Off sublamination in the ferret [4]. Horizontal sections through the dLGN were cut at 40 μm. Sections were rinsed in 0.1 M phosphate buffer (PB) and blocked for 4 hours at room temperature in 10% normal donkey serum (Jackson ImmunoResearch Laboratories, West Grove, PA, USA) with 0.3% Triton X-100 in 0.1 M PB. Sections were incubated in TED15 primary antibody overnight (1:1,000; gift of Dr Eldon Geisert, Hamilton Eye Institute, University of Tennessee). Sections were washed overnight in 0.1 M PB and transferred to biotinylated secondary antibody solution (1:200 donkey anti-mouse IgM, Vector Laboratories) for 2 h at room temperature. Sections were washed 5 × 30 minutes in 0.1 M PB and transferred to 0.1 M PB containing the peroxidase conjugate from the Vectastain ABC kit (Vector Laboratories). Sections were rinsed 5 × 30 minutes in 0.1 M PB and briefly immersed (2 to 3 minutes) in a solution of 0.02% 3-3' diaminobenzidine-4HCl (DAB, Sigma-Aldrich) and 0.003% hydrogen peroxide. After staining, sections were mounted on gelatinized glass slides, dehydrated in increasing concentrations of ethanol, cleared in xylene and cover-slipped with DPX medium (Sigma-Aldrich).

Five to seven sections from each side of the dorsal thalamus were imaged as in [3]. For both control and Ferret VAChT-SAP-treated cases, images were grayscaled, inverted, and an unsharp mask filter was applied at 500% with a radius of 50 pixels and threshold of zero. Input levels were maximized for each image. This uniform image transformation was applied identically to all images, control and treated cases. The boundaries of the contralateral and ipsilateral laminae were manually outlined and the area fraction corresponding to positive DAB signal in the intersublaminar space was automatically calculated using ImageJ [57]. N = 3 saline controls (6 dLGNs); N = 5 Ferret VAChT-SAP animals (10 dLGNs).

### Single-unit electrophysiological recording

Animals treated at P9/P10 with either saline or Ferret VAChT-SAP were allowed to recover for >100 days following eye injections. Electrophysiological recordings were performed as in [14]. Briefly, animals were anesthetized using a mixture of acepromazine (0.4 mg/kg) and ketamine (40 mg/kg intramuscularly) and placed in a modified kitten stereotax. Animals were intubated and mechanically ventilated to maintain anesthesia using 2% inhalant isoflurane in oxygen (volume and rate to maintained peak inspiratory pressure at 1.5 kPa and end-tidal carbon dioxide at 3.5 to 5%). End-tidal carbon dioxide and core body temperature were monitored throughout the experiment. Prior to reverse correlation white-noise receptive field mapping, animals were paralyzed with vecuronium bromide (0.2 mg/kg/h intravenously). A 4 × 4 mm craniotomy was made over the dLGN, lacquered tungsten electrodes (Micro Probe, Potomac, MD, USA; impedance 5 or 1.5 MΩ) were advanced through the depth of the dLGN using a microdrive, and visual responses were recorded every 100 to 200 μm. To map the organization of On- and Off-center cells, dLGN activity was assessed with an audio monitor and oscilloscope, and receptive fields were mapped onto a tangent screen using a handheld light. Electrode penetrations were spaced in a 200-μm grid across the rostrocaudal and mediolateral extent of the dLGN. To obtain peristimulus time histograms and receptive field maps, visual stimuli were created using a VSG 2/5 visual stimulator (Cambridge Research Systems, Rochester, UK) and displayed on a Sony monitor with a mean luminance of 40 to 50 candelas/m^2^. Neuronal responses were amplified, filtered, and recorded to a PC equipped with a Power 1401 data acquisition interface and the Spike 2 software package (Cambridge Electronic Design, Cambridge, UK). Spike isolation was based on waveform analysis (on-line and off-line) and presence of a refractory period, as indicated by the autocorrelogram. Visual stimuli were created with a VSG2/5 visual stimulus generator (Cambridge Research Systems). Receptive fields of dLGN neurons were mapped quantitatively by reverse correlation using pseudorandom spatio-temporal white-noise stimuli (m-sequences) [58]. The white-noise stimulus (100% contrast) consisted of a 16 × 16 grid of squares (pixels) that were white or black one half of the time during an m-sequence of length 2^15^-1. The size of individual pixels was 1.4° squared. The speed of presentation was varied at 1, 3, 5, or 7 frames per term. At 1 frame per term, the stimulus was updated every 15.625 ms; at 3 frames per term, every 46.875 ms; at 5 frames per term, every 78.125 ms; and at 7 frames per term, every 109.375 ms. Receptive field areas of dLGN neurons were quantified as in [59].

Prior to each experiment, animals were injected intraocularly with anterograde tracers to visualize retinogeniculate afferents (see above). Upon termination of each experiment, animals were perfused with 0.9% saline and 4% paraformaldehyde, and dLGN and retinal tissue was collected. Injection of anterograde tracers did not impact the receptive field structure of individual dLGN neurons as evidenced by control experiments confirming RF stability of single identified neurons pre and post eye-injection (data not shown). All saline animals exhibited normal anatomical separation of retinogeniculate afferents into On and Off sublaminae while the retinal afferent free margin indicative of such segregation was absent in each Ferret VAChT-SAP recovery case. Visualization of electrode tracks confirmed that our penetrations were confined to the dLGN in all cases. ChAT immunohistochemistry on dissected retinae confirmed the ablation of SACs in adult animals treated with Ferret VAChT-SAP. N = 2 saline control ferrets; N = 6 Ferret VAChT-SAP recovery ferrets.

### Nearest neighbor analysis

Nearest neighbor analysis of On and Off organization in the dLGN was performed as in [60]. A binomial model of the formula p(same) = p(On)^2 ^+ p(Off)^2 ^was used to calculate the likelihood of encountering neighboring cells of the same center-type given a randomization of the observed numbers of On and Off cells in the data. In this randomization, p(same) is the probability of two neighboring cells being of identical sign (On/On or Off/Off), p(On) = (Number of On-center cells)/(Total number of (On + Off)-center cells in the recorded population), and p(Off) = (Number of Off-center cells)/(Total number of (On + Off)-center cells in the recorded population). After calculating p(same) based on the observed frequencies of On and Off cells, the predicted number of neighboring cells of the same center-type in a randomization of the data can be calculated by multiplying p(same) by the total cell number (On + Off).

## Abbreviations

AC: amacrine cell; CI: correlation index; dLGN: dorsal lateral geniculate nucleus; HC: horizontal cell; ISI: interspike interval; MEA: multi-electrode recording array; P: postnatal day; PB: phosphate buffer; RF: receptive field; RGC: retinal ganglion cell; SAC: starburst amacrine cell; SEM: standard error of the mean; VAChT: vesicular acetylcholine transporter.

## Competing interests

The authors declare that they have no competing interests.

## Authors' contributions

CMS cloned the ferret *VAChT *gene used for generating the immunotoxin. CMS performed eye injections, tract tracing, and immunohistochemistry. CMS and CS performed retinal multi-electrode array recording experiments and data analysis. CMS performed dLGN electrophysiological recording experiments and data analysis. CMS and BC designed the study and wrote the manuscript.

## Supplementary Material

Additional file 1 **Timeline of experimental manipulation relative to development of On/Off sublaminae in the ferret dLGN**. **(A) **In the ferret, eye-specific segregation is complete by postnatal day 10 (P10) and On/Off sublamination occurs between P15 and P25 corresponding to the period of ABAKAN expression in the emerging intersublaminar spaces. **(B) **We disrupted spontaneous retinal activity by injection of Ferret VAChT-SAP at P9/P10, after completion of eye-specific segregation but prior to On/Off segregation and ABAKAN expression in the intersublaminar spaces. We then evaluated the expression of ABAKAN at P25, the time point in development where ABAKAN normally appears in the intersublaminar spaces between On/Off sublaminae. **(C) **Anterograde labeling of eye-specific retinogeniculate afferents was performed at the same age. Separate cohorts were treated identically and then allowed a period of normal vision after eye-opening. Electrophysiological recording in the dLGN and retinogeniculate tract tracing were then performed in adulthood (C). Click here for file
