# Supplementary figures and images for: Activity-dependent disruption of intersublaminar spaces and ABAKAN expression does not impact functional on and off organization in the ferret retinogeniculate system
Source: Neural Dev. 2011 Mar 14;6:7. doi: 10.1186/1749-8104-6-7 (PMC3065403; doi:10.1186/1749-8104-6-7)

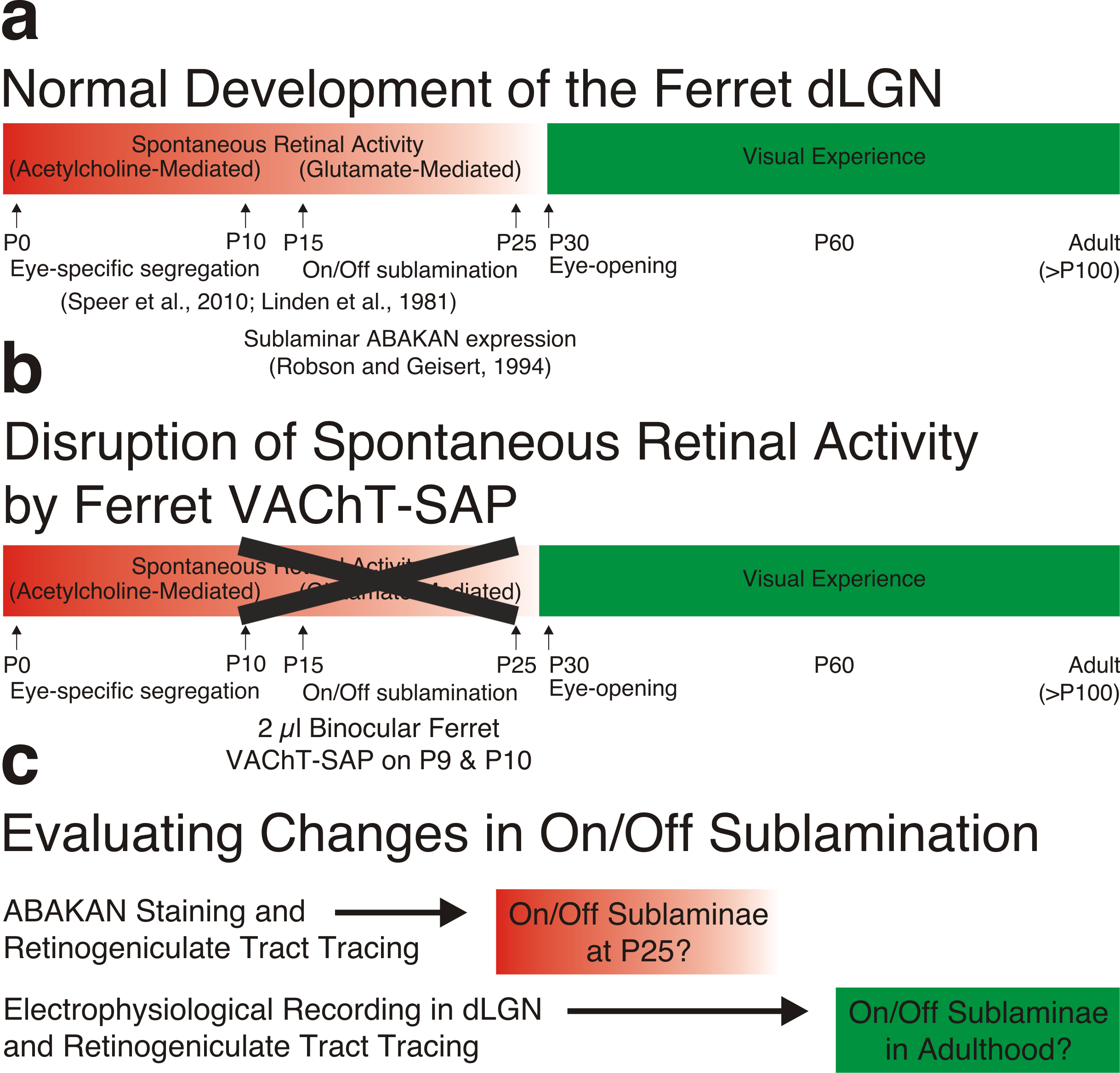

Supplement: Additional file 1 — Timeline of experimental manipulation relative to development of On/Off sublaminae in the ferret dLGN. (A) In the ferret, eye-specific segregation is complete by postnatal day 10 (P10) and On/Off sublamination occurs between P15 and P25 corresponding to the period of ABAKAN expression in the emerging intersublaminar spaces. (B) We disrupted spontaneous retinal activity by injection of Ferret VAChT-SAP at P9/P10, after completion of eye-specific segregation but prior to On/Off segregation and ABAKAN expression in the intersublaminar spaces. We then evaluated the expression of ABAKAN at P25, the time point in development where ABAKAN normally appears in the intersublaminar spaces between On/Off sublaminae. (C) Anterograde labeling of eye-specific retinogeniculate afferents was performed at the same age. Separate cohorts were treated identically and then allowed a period of normal vision after eye-opening. Electrophysiological recording in the dLGN and retinogeniculate tract tracing were then performed in adulthood (C). [file 1749-8104-6-7-S1.TIFF]
